# Adaptation and validation of the *Adherence Barriers Questionnaire for HIV Patients on Antiretroviral Therapy* (ABQ-HIV) for the Brazilian context

**DOI:** 10.1590/0102-311XEN006324

**Published:** 2024-09-16

**Authors:** Míria Dantas Pereira, Sabrina Müeller, Victor Santana Santos

**Affiliations:** 1 Universidade Federal de Sergipe, Aracaju, Brasil.; 2 University of Wismar, Wismar, Germany.; 3 Universidade Federal de Sergipe, Lagarto, Brasil.

**Keywords:** Hospital Care, Hospital Information Systems, Benchmarking, Indicators (Statistic), Unified Health System, Assistência Hospitalar, Sistemas de Informação Hospitalar, Benchmarking, Indicadores (Estatística), Sistema Único de Saúde, Atención Hospitalaria, Sistemas de Información en Hospital, Benchmarking, Indicadores (Estadística), Sistema Único de Salud

## Abstract

Despite significant advancements in antiretroviral therapy (ART) for HIV, adherence remains a challenge. While Brazil has validated scales for treatment adherence, few assess treatment adherence barriers. This underscores the necessity for validated questionnaires on adherence barriers to identify patient-specific challenges and enhance strategies for ART adherence. This study aimed to adapt and validate the *Adherence Barriers Questionnaire for HIV Patients on Antiretroviral Therapy* (ABQ-HIV), a 17-item questionnaire assessing the adherence barriers to ART, for the Brazilian context and to evaluate its psychometric properties in HIV patients. A methodological study on the psychometric properties and factorial structure of ABQ-HIV was conducted. The study followed seven steps: consent of the original authors, two translations, synthesis of the translations, expert committee, back-translation, pre-test, and reliability test. A high content validity index (0.93) was achieved with the expert committee. The study sample consisted of 230 adults with HIV, with 37.0 (29.3-45.0) years as the median age (IQR), and 52.2% were male. The exploratory factor analysis with a three subscales structure of 17 items showed good interpretability (Bartlett’s sphericity (1167.2 [136]; p < 0.001) and Kaiser-Meyer-Olkin = 0.602) and internal consistency (α = 0.76; Ω = 0.76). The fit indicators were satisfactory (χ^2^ = 89.931; df = 88; p > 0.005; RMSEA = 0.010; RMSR = 0.07; CFI = 0.996; GFI = 0.940; AGFI = 0.907; NNFI = 0.995). The Brazilian version of ABQ-HIV is a potential instrument for identifying specific barriers to adherence to ART in adults living with HIV in Brazil.

## Abstract (pt)

Apesar dos avanços significativos na terapia antirretroviral (TARV) para o HIV, a adesão continua sendo um desafio. Embora o Brasil tenha escalas validadas para a adesão ao tratamento, poucas avaliam as barreiras de adesão ao tratamento. Isso ressalta a necessidade de questionários validados sobre as barreiras de adesão para identificar os desafios específicos dos pacientes e aprimorar as estratégias de adesão à TARV. Este estudo teve como objetivo adaptar e validar o *Adherence Barriers Questionnaire for HIV Patients on Antiretroviral Therapy* (ABQ-HIV), um questionário de 17 itens que avalia as barreiras de adesão à TARV, para o contexto brasileiro e avaliar suas propriedades psicométricas em pacientes com HIV. Foi realizado um estudo metodológico sobre as propriedades psicométricas e a estrutura fatorial do ABQ-HIV. O estudo seguiu sete etapas: consentimento dos autores originais, duas traduções, síntese das traduções, comitê de especialistas, retrotradução, pré-teste e teste de confiabilidade. Um comitê de especialistas obteve um alto índice de validade de conteúdo (0,93). A amostra do estudo foi de 230 adultos com HIV, com idade média (IQR) de 37,0 (29,3-45,0) anos, e 52,2% eram homens. A análise fatorial exploratória com uma estrutura de três subescalas de 17 itens mostrou boa interpretabilidade (esfericidade de Bartlett (1167,2 [136]; p < 0,001) e Kaiser-Meyer-Olkin = 0,602) e consistência interna (α = 0,76; Ω = 0,76). Os indicadores de ajuste foram satisfatórios (χ^2^ = 89,931; df = 88; p > 0,005; RMSEA = 0,010; RMSR = 0,07; CFI = 0,996; GFI = 0,940; AGFI = 0,907; NNFI = 0,995). A versão brasileira do ABQ-HIV é um instrumento em potencial para identificar barreiras específicas à adesão à TARV em adultos vivendo com HIV no Brasil.

## Abstract (es)

A pesar de los avances significativos de la terapia antirretroviral (TARV) para el VIH, la adherencia sigue siendo un desafío. Aunque Brasil ha validado las escalas de adherencia al tratamiento, pocas escalas evalúan las barreras de adherencia al tratamiento. Esto subraya la necesidad de cuestionarios validados sobre las barreras de adherencia para identificar los desafíos específicos de los pacientes y mejorar las estrategias de adherencia a la TARV. Este estudio tuvo como objetivo adaptar y validar el *Adherence Barriers Questionnaire for HIV Patients on Antiretroviral Therapy* (ABQ-VIH), un cuestionario de 17 ítems que evalúa las barreras para la adherencia a la TARV para el contexto brasileño, y evaluar sus propiedades psicométricas en pacientes con VIH. Se realizó un estudio metodológico sobre las propiedades psicométricas y la estructura factorial del ABQ-VIH. Este estudio consistió en siete etapas: consentimiento de los autores originales, dos traducciones, síntesis de traducciones, comité de expertos, retrotraducción, prueba previa y prueba de confiabilidad. Un comité de expertos obtuvo un alto índice de validez de contenido (0,93). La muestra del estudio estuvo conformada de 230 adultos con VIH, con una edad media (IQR) de 37,0 (29,3-45,0) años, y el 52,2% de ellos son del sexo masculino. El análisis factorial exploratorio con una estructura de tres subescalas de 17 ítems mostró una buena interpretabilidad (esfericidad de Bartlett (1167,2 [136]; p < 0,001) y Kaiser-Meyer-Olkin = 0,602) y consistencia interna (α = 0,76; Ω = 0,76). Los indicadores de ajuste fueron satisfactorios (χ^2^ = 89,931; df = 88; p > 0,005; RMSEA = 0,010; RMSR = 0,07; CFI = 0,996; GFI = 0,940; AGFI = 0,907; NNFI = 0,995). La versión brasileña de ABQ-VIH es una potencial herramienta para identificar barreras específicas en la adherencia a la TARV en adultos que viven con VIH en Brasil.

## Introduction

The human immunodeficiency virus (HIV), the etiological agent responsible for the AIDS, is one of the greatest obstacles to global public health due to its pandemic nature and associated severity ^1^. HIV infection compromises the function of CD4+ cells, which are responsible for defending the immune system ^2^. Without treatment, progression to AIDS can lead to significant and potentially lethal clinical complications ^2^.

AIDS is recognized as one of the most fatal infectious diseases recorded in history, with approximately 40.4 million global deaths ^3^. By the end of 2022, around 39 million people were living with HIV/AIDS worldwide ^3^. In Brazil, the HIV/AIDS Epidemiological Bulletin reports 1,124,063 AIDS cases in 43 years (1980 to June 2023) ^4^. Between 2007 and June 2023, the country recorded 489,594 cases of HIV infection, 21.3% of which occurred in the Northeast Region, which has the second-highest case detection rate in the country ^4^.

Antiretroviral therapy (ART) transformed HIV infection into a manageable chronic condition, significantly reducing deaths related to the disease globally ^1^. According to World Health Organization (WHO) guidelines ^5^, ART is recommended for all people living with HIV (PLHIV), aiming at benefits such as effective immune reconstitution, control of viral replication, reduction of transmission, and AIDS mortality ^6^. Treatment adherence of less than 85% can increase drug resistance and disease progression ^7^.

Studies have unveiled multiple barriers that significantly impact adherence to ART. These include intrapersonal factors such as age (younger populations), alcohol/drug use, low schooling, anxiety/depression disorders, and forgetfulness ^8^; social/environmental factors such as HIV stigma/discrimination and fear of disclosure ^9^; drug-related factors such as access, side effects, and complexity of the regimen ^10^; and health system factors such as the quality of the physician-patient relationship and the level of education/information ^11^.

Understanding the barriers to adherence to treatment by PLHIV is crucial for the efficacy of ART and for targeting effective interventions. Identifying predictive factors to manage unsuppressed viral loads and avoid costly resistance testing ^12^. Validated self-report questionnaires are valuable for capturing patient perspectives on treatment and are a practical, cost-effective approach that focuses on knowledge of the disease, attitude towards drugs, and management of side effects ^13^.

Specific questionnaires tailored to assess adherence among individuals with HIV/AIDS receiving ART are lacking in Brazil ^14^ ^,^ ^15^ ^,^ ^16^ ^,^ ^17^. A recent scoping review ^14^ identified only three validated instruments in Brazil to assess adherence to antiretroviral therapy in PLHIV: (a) *Questionnaire for Assessment of Adherence to Antiretroviral Treatment* ^15^; (b) WebAd-Q questionnaire ^16^; and (c) *Self-efficacy Scale for Adherence to Antiretroviral Treatment in Children and Adolescents with HIV/AIDS* ^17^. However, existing validated questionnaires primarily focus on treatment adherence per se, focusing on medication intake ^14^. A validated questionnaire specifically designed to explore the barriers to adherence among people living with HIV/AIDS in Brazil is unavailable. This gap in assessment tools impedes a comprehensive understanding of the multifaceted challenges individuals face in adhering to ART regimens, thus highlighting the urgent need for the development and validation of such instruments within the Brazilian context.

Given this, we opted for the *Adherence Barriers Questionnaire for HIV Patients on Antiretroviral Therapy* (ABQ-HIV) ^18^, which was adapted from a German study involving 370 participants. The ABQ-HIV comprises 17 items that investigate the barriers to adherence affecting patient behaviour concerning ART. Its effectiveness has already been proven for other chronic conditions ^19^ ^,^ ^20^. This study aimed to adapt and validate the ABQ-HIV for the Brazilian context and to evaluate its psychometric properties in individuals with HIV. The overarching goal is to provide a practical and easy tool for understanding and improving adherence to ART in Brazil.

## Method

### Study design

This is a methodological, cross-sectional, and quantitative study carried out to translate and adapt the ABQ-HIV for the Brazilian context. This study was conducted according to the COSMIN methodology (COnsensus-based Standards for the selection of health Measurement INstruments), which is considered a standard guide for assessing the psychometric quality of health measurement questionnaires ^21^. The study phases were: (a) consent from the authors of the original questionnaire; (b) translation by two independent translators; (c) synthesis of the translations; (d) expert committee; (e) back-translation; (f) pre-test; and (g) assessment of reliability in the target population.

### Study setting and participants

The study was conducted with a sample of PLHIV who were being attended at the sexually transmitted infection (STI), HIV, and AIDS Specialized Care Service of the Aracaju Medical Specialties Center. This center is the main point of reference for PLHIV in the state of Sergipe, providing services to approximately 6,185 patients on ART ^22^.

To be included in the study, PLHIV had to meet the following criteria: (a) positive HIV serology; (b) aged 18 years or older; (c) use of ART for at least one year. Individuals with severe neuropsychological impairment or psychosis were excluded from the study, as were those who were unable to complete the interviews required for the study.

### Sample size

Sample sizes were defined for each phase of the study. To guarantee the reliability of the questionnaire, 126 individuals were required, based on a 0.8 minimum item-total correlation, a 0.15 margin of error, an 5% alpha, and 95% power. To structure the questionnaire, the sample size was calculated using the Inverse Square Root ^23^ method:

N^≥z1-α2+zββmin2

Where, $\hat{N}$ is the sample size, $z_{1-\frac{\alpha }{2}}$ is the normal distribution score associated with the significance level α and *z*_*β*_ is the normal distribution score associated with the power of the test *β* and *|β|*_*min*_ is the effect between factors. Assuming 5% significance, 95% power, and a 0.25 minimum effect, 208 participants were needed.

### Instruments

Sociodemographic data (such as age, sex, marital status) and clinical data (such as CD4+ count, viral load, ART regimen) were collected using a pre-defined questionnaire designed for the study. Data on clinical profile, time of use and ART regimen were confirmed by retrieving the medical records.

The ABQ-HIV ^18^, a German questionnaire, is an instrument with 17 Likert-scaled items divided into three subscales (unintentional barriers, barriers associated with knowledge of the disease/treatment, and intentional barriers) to identify barriers to adherence to treatment in PLHIV. The questionnaire showed good internal consistency (α = 0.708) and convergent validity (-0.422; p < 0.001) when compared to the 8-item *Morisky Medication Adherence Scale* (MMAS-8). Responses range from 1 to 4 from “strongly agree” to “strongly disagree,” and a total score above 28 indicates a higher risk of significant barriers to adherence. The higher the score, the greater the barrier burden and, thus, the associated risk of non-adherence.

### Translation, cross-cultural adaptation, and pre-test stages

For the cross-cultural adaptation ^21^ ^,^ ^24^ of the ABQ-HIV, we first obtained authorization from the authors. The original German version was translated into Brazilian Portuguese by two independent and blinded translators. The first translation (T1) was carried out by a translator with expertise in the health field and adopted a technical perspective, whereas the second translation (T2) was performed by a professional translator without expertise in health and adopted an idiomatic approach. Both translations encompassed the title, instructions, response options, and the 17 items of the ABQ-HIV questionnaire. A synthesized version (T1-2) was elaborated, integrating the most relevant aspects of the preceding translations.

Professionals with expertise in HIV were recruited through the Lattes Platform of the Brazilian National Research Council (CNPq) to assess construct validity. Health professionals with clinical experience of 10 years or more in treating HIV patients and carrying out validation studies were selected. A total of 15 judges took part in the study: five infectious disease doctors, four pharmacists, three nurses, one psychologist, one social worker, and one speech-language therapist. All the experts assessed the suitability and relevance of the items using a four-point ordinal scale (1 “not relevant”, 2 “slightly relevant”, 3 “quite relevant”, and 4 “highly relevant”).

The judges evaluated the items, recommending reformulations and additions for Brazilian sociocultural adequacy, aiming for clarity and relevance. The agreement was quantified by the content validity index (CVI), with an acceptable minimum of 0.80 and preferably 0.90. The CVI was calculated as the sum of answers 3 and 4 divided by the total number of answers, reflecting the proportion of agreement on relevance ^25^.

**Figure 1:**
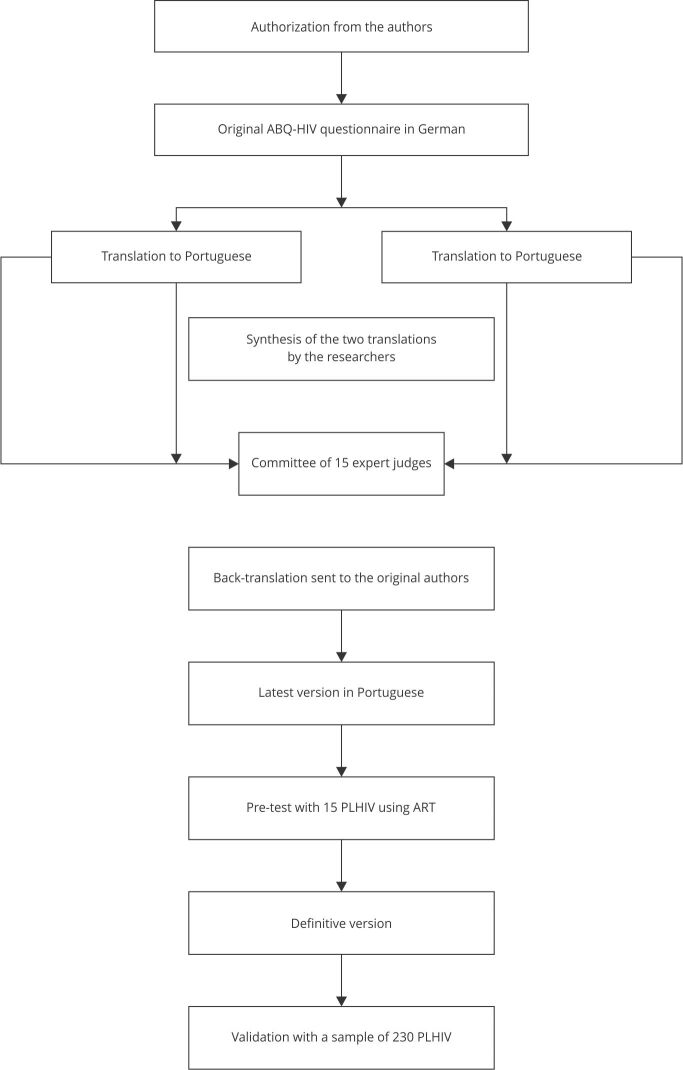
Flowchart of the adaptation and validation process of the *Adherence Barriers Questionnaire for HIV Patients on Antiretroviral Therapy* (ABQ-HIV).

The modified questionnaire underwent a back-translation process, wherein a native German speaker translated it from Portuguese to German. Subsequently, the translated version was forwarded to the original authors for evaluation of semantic equivalence and to obtain their consent. Finally, a pre-test involving 15 individuals from the target population was carried out to identify any uncertainties or challenges. Since no notable issues emerged during the test, the Portuguese version was considered definitive. Figure 1 shows the methodological process.

PLHIV: people living with HIV.

### Data collection procedures

Participants were selected based on eligibility criteria, and data collection occurred from October 2022 to January 2023. The process included structured interviews and the application of the ABQ-HIV Brazilian version. With their consent, the participants completed supervised questionnaires in individual 15-minute sessions, placing emphasis on the completeness of their answers and ensuring anonymity.

### Data analysis

Categorical variables were described using frequencies and percentages. Normality assumptions were assessed using the Shapiro-Wilk test, and homoscedasticity was evaluated using the Levene test. We conducted nonparametric tests to verify the significance of the distributions between the study variables, as most datasets had a skewed distribution.

The adequacy of the ABQ-HIV questionnaire was assessed using the Kaiser-Meyer-Olkin (KMO) test (expected to be > 0.5) ^26^ and Bartlett’s test of sphericity (expected to be p < 0.05) ^27^. The unidimensionality was assessed using Unidimensional Congruence (UniCo) (expected to be > 0.95), explained common variance (ECV) (expected to be > 0.85), and mean of item residual absolute loadings (Mireal) (expected to be < 0.30) tests ^28^ ^,^ ^29^. Exploratory factor analysis (EFA) was used to examine the factor structure of the ABQ-HIV questionnaire. The EFA used a polychoric matrix and the robust diagonally weighted least squares (RDWLS) method. The number of retained subscales was determined through the parallel analysis (PA) method ^30^ employing random permutation of observed data, along with Robust Promin rotation ^31^ and empirical LOSEFER correction ^32^.

The stability of the subscales of the adapted questionnaire was assessed using the Generalized G-H Index, with G-H values ranging from 0 to 1. High G-H values (> 0.80) suggest a well-defined latent variable likely to be stable across studies, whereas low values indicate a poorly defined and likely unstable latent variable across studies ^33^.

To confirm the fit of the model, indices such as chi-square (χ^2^), root mean square error of approximation (RMSEA), comparative fit index (CFI), non-normed fit index (NNFI), and Tucker-Lewis index (TLI) were used. We have adopted the following threshold for the adequacy of the model: RMSEA < 0.08, CFI > 0.90, and TLI > 0.90 ^34^. Reliability was evaluated using Cronbach’s alpha (α > 0.70) and McDonald’s omega (Ω > 0.70).

The descriptive analyses were carried out using Stata version 14.0 (https://www.stata.com) and the AFE using Factor Analysis Application (version 14.04.01). Statistical significance was set at p < 0.05.

### Ethical aspects

The study was approved by the Human Research Ethics Committee of the Federal University of Sergipe (CAAE: 51176221.5.0000.5546, protocol n. 5.036.636). All investigations were conducted according to the Declaration of Helsinki and *Resolution n. 466/2012* of the Brazilian National Health Council. Informed written consent was obtained from the participants.

## Results

### Translation, committee of expert judges, back-translation, and pre-testing

A comparison between translation 1 and translation 2 revealed minimal stylistic discrepancies, which did not compromise fidelity to the original content.

The content analysis revealed that 15 out of 17 items had a CVI > 0.80, with 10 items achieving a perfect 1.00 CVI. This indicates a high level of agreement regarding the equivalence of the content assessed. However, two items (#5 and #8) fell below the established threshold, with 0.46 and 0.73 CVI values, respectively, requiring adjustments to enhance objectivity and comprehension in the Brazilian context. These results are shown in Table 1.

**Table 1:** Agreement values for the content validity index (CVI). Aracaju, Sergipe State, Brazil, 2023.

Item	Description of the summary version	CVI
1	“I understand well what my doctor, a nurse or a pharmacist have explained to me so far”	1.00
2	“I can name my medicines and their uses without hesitation”	0.80
3	“I trust my doctor and agree on my treatment plan with him”	0.93
4	“My medications only help if I take them absolutely regularly, as recommended”	1.00
5	“All medicine is poison. If possible, one should avoid taking medicine.”	0.46
6	“In principle, I feel healthy. So sometimes I’m not sure if I should be taking my medications daily”	0.86
7	“I automatically take my medications every day at a certain time or at certain times (e.g. during meals, at bedtime)”	0.93
8	“Additional payments for medicines represent a real burden for me”	0.73
9	“Basically, I feel uncomfortable when other people notice my medication intake”	0.93
10	“I often forget things in my daily life”	1.00
11	“In general, I often feel low; sometimes I also feel discouraged and depressed”	1.00
12	“I often have difficulty taking my medications (e.g., swallowing, dividing the pills, opening the packaging) or it is difficult to adhere to the conditions for taking the medications (e.g., fasting, not eating certain foods and/or or alcohol)”	1.00
13	“In particular, I find it difficult to follow my treatment when I’m away from home (e.g. at weekends, on business trips or on vacation)”	1.00
14	“I have great support from family and/or friends who I can talk to at any time and who I can turn to for help”	1.00
15	“I’m very afraid of the side effects of my medications”	1.00
16	“If I notice/noticed side effects related to my medications, I would talk to my doctor about them as soon as possible”	1.00
17	“If I notice/noticed side effects from my medicines, I would first stop/quit taking them or take/take less of them”	1.00

Item #5 was modified to replace ambiguous terms with clearer expressions, ensuring the original meaning. As for item #8, alternatives were introduced to encompass additional barriers to ART access, such as the economic burden of treatment, given the context of free treatment provided by the Brazilian Unified National Health System (SUS) in Brazil. These adjustments, guided by judges’ recommendations, led to a 0.93 overall CVI for the questionnaire. After back-translation into German and approval by the authors of the original questionnaire, the pre-final Portuguese version was tested by the target audience, who reported no difficulties. The final Portuguese version of the ABQ-HIV questionnaire, adapted for the Brazilian context, is presented in the Supplementary Material (https://cadernos.ensp.fiocruz.br/static//arquivo/suppl-e006324_1322.pdf).

### Validation of the ABQ-HIV Brazilian version

#### Characterization of the sample

In total, 230 adults living with HIV were included and fully completed the ABQ-HIV. Table 2 shows the sociodemographic characteristics of the individuals included. Ages ranged from 19 to 78 years, with a median of 37.0 years (interquartile range, IQR: 29.3-45.0). Most participants were male (120, 52.2%), self-declared as brown ethnicity (112, 48.7%), living in urban areas (163, 70.9%), single (160, 69.6%), earning 1-2 Brazilian minimum wages (108, 47%), with completed secondary education (67, 29.1%).

**Table 2:** Sociodemographic characteristics of the participants included in the study. Aracaju, Sergipe State, Brazil, 2023.

Variable	n (%)
Age (years) [median (IQR)]	37.0 (29.3-45.0)
Sex	
Male	120 (52.2)
Female	110 (47.8)
Ethnicity	
White	62 (27.0)
Brown	112 (48.7)
Black	50 (21.7)
Indigenous	5 (2.2)
Yellow	1 (0.4)
Area of residence	
Urban	163 (70.9)
Rural	67 (29.1)
Working	
Yes	104 (45.2)
No	126 (54.8)
Marital status	
Single	160 (69.6)
Married	34 (14.8)
Stable union	17 (7.4)
Separate	3 (1.3)
Divorced	8 (3.5)
Widower	8 (3.5)
Sexual orientation	
Heterosexual	138 (60.0)
Homosexual	74 (32.2)
Bisexual	7 (3.0)
Ignored	11 (4.8)
Income (minimum wage)	
No income	59 (25.7)
< 1	38 (16.5)
1-2	108 (47.0)
2-5	21 (9.1)
> 5	2 (0.9)
Ignored	2 (0.9)
Education	
Illiterate	10 (4.3)
Incomplete elementary education	66 (28.7)
Complete primary education	18 (7.8)
Incomplete high school	24 (10.4)
Complete high school	67 (29.1)
Incomplete higher education	25 (10.9)
Complete higher education	20 (8.7)

IQR: interval interquartile range.

The median (IQR) time since starting ART was 5.0 (IQR: 2.0-8.0) years. One hundred and twenty-four (53.9%) participants reported using condoms in all sexual relations in the last six months. Most individuals reported previous (104, 45.2%) partners as their exposure to HIV; and 88 (38.3%) were unaware of their main source of exposure to HIV. The rapid test (point-of-care) and routine examinations were the main methods of HIV diagnosis, with 117 (50.9%) and 90 (39.1%), respectively. The most common treatment regimen was the combination of Tenofovir + Lamivudine + Dolutegravir (126, 54.8%) and Lamivudine + Tenofovir + Efavirenz (55, 23.9%), both prescribed as a single-dose regimen. Most participants had a CD4 count of 500cells/mm^3^ or higher (133, 57.8%). The viral load was 40 copies or less in 128 (55.7%) of participants. (Table 3).

**Table 3:** Clinical characteristics of the participants included in the study. Aracaju, Sergipe State, Brazil, 2023.

Variable	n (%)
Diagnosis time (years) [median (IQR)]	5.0 (2.0-9.0)
Time of ART use (years) [median (IQR)]	5.0 (2.0-8.0)
Condom use	
Always	124 (53.9)
Sometimes	62 (27.0)
Never	27 (11.7)
Not applicable	8 (3.5)
Ignored	9 (3.9)
HIV exposure	
HIV-positive current partner	28 (12.2)
HIV-positive previous partner	104 (45.2)
Vertical transmission	6 (2.6)
Do not know	88 (38.3)
Ignored	4 (1.7)
Diagnosis	
Rapid test	117 (50.9)
Routine examination	90 (39.1)
During hospitalization	19 (8.3)
Blood bank	3 (1.3)
Ignored	1 (0.4)
Current ART regimen	
Tenofovir + Lamivudine + Dolutegravir	126 (54.8)
Lamivudine + Tenofovir + Efavirenz	55 (23.9)
Tenofovir + Lamivudine + Atazanavir/ Ritonavir	17 (7.4)
Lamivudine + Tenofovir + Darunavir/ Ritonavir	15 (6.5)
Zidovudine + Efavirenz + Tenofovir	4 (1.7)
Others	13 (5.7)
Adverse effects of ART	
Yes	83 (36.1)
No	147 (63.9)
CD4 count (cells/mm^3^)	
≥ 500	133 (57.8)
499 to 200	80 (34.8)
< 200	17 (7.4)
Viral load (copies)	
≤ 40	128 (55.7)
40 to 10,000	82 (35.7)
10,000 to 100,000	17 (7.4)
100,000 to 1,000,000	3 (1.3)
Self-assessment of ART adherence	
Very good	97 (42.2)
Good	101 (43.9)
Regular	23 (10.0)
Bad	9 (3.9)

ART: antiretroviral therapy; IQR: interval interquartile range.

#### Factor structure and psychometric properties of the ABQ-HIV

The data matrix showed favorable indicators (KMO = 0.602; IC95: 0.194-0.499 and Bartlett's Test of Sphericity = 1167.2 [136]; p < 0.001). The hypothesis of unidimensionality was rejected based on the values obtained for UniCo (0.68) and ECV (0.67), whereas the Mireal criterion (0.28) was the only one to suggest such a possibility.

The PA revealed two subscales for the Brazilian version of the ABQ-HIV questionnaire, with variations of 26.45% for Factor#1 and 12.62% for Factor#2. These results differ from the three subscales structure proposed in the original version of the ABQ-HIV questionnaire ^18^. For this reason, testing the number of subscales in the questionnaire followed the theoretical model on which it was built, i.e., with three subscales. The data matrix was interpreted using the weighted Varimax method, which was used to extract orthogonal factors, i.e., not correlated with each other.

The subsequent factor analysis, fixing three factors, revealed a factor structure with subscales that can be identified as unintentional barriers, barriers associated with knowledge of the disease/treatment, and intentional barriers. The variance explained by each extracted subscale was 26.45%, 12.62%, and 8.69%, respectively.

The factor loadings of the items (Table 4) showed saturations above 0.30, ranging from 0.32 to 0.69. The non-intentional factor comprised six items (4, 6, 7, 12, 13, 17), with saturations ranging from 0.33 (Item 7) to 0.62 (Item 13). The knowledge factor added seven items (2, 5, 8, 9, 10, 11, 15), with saturations ranging from 0.32 (Item 8) to 0.69 (Item 11). The intent factor included four items (1, 3, 14, 16), with saturations ranging from 0.41 (Item 1) to 0.51 (Item 16).

**Table 4:** Subscales identified through factor analysis. Aracaju, Sergipe State, Brazil, 2023 (N = 230).

Item	Subscale 1 “Unintentional”	Subscale 2 “Knowledge”	Subscale 3 “Intentional”
Loading value (95%CI)	Loading value (95%CI)	Loading value (95%CI)
Item 1: “I understand well what my doctor, nurse or pharmacist explained to me about my medication treatment”			0.413 (-0.058-1.716)
Item 2: “I can name the names of my medications, what they are for and how I should take them”		0.344 (-0.003-1.123)	
Item 3: “I trust my doctor and agree on my treatment plan with him”			0.477 (0.056-7.966)
Item 4: “My medications only help if I take them regularly, as recommended”	0.412 (-0.056-22.001)		
Item 5: “Generally, any medicine is in some way harmful and therefore you should avoid taking medicines whenever possible”		0.455 (0.207-5.180)	
Item 6: “In principle, because I do not feel sick, sometimes I’m not sure if I should take my medicines daily”	0.521 (0.165-2.054)		
Item 7: “I take my medications every day automatically at a certain time or on certain occasions (e.g. during meals, at bedtime)”	0.339 (-0.071-13.605)		
Item 8: “Additional expenses to receive my medicines (e.g. transportation, eating out) represent a real difficulty for me”		0.327 (-0.017-0.643)	
Item 9: “I feel uncomfortable when other people notice that I am taking medication”		0.664 (0.535-6.476)	
Item 10: “I often forget things in my daily life”		0.599 (0.355-2.012)	
Item 11: “I often feel low; sometimes also discouraged and depressed)”		0.697 (0.416-7.499)	
Item 12: “I have difficulty taking my medications (e.g. swallowing, dividing pills, opening packaging) or it is difficult to follow medication recommendations (e.g. fasting, not eating certain foods and/or alcohol)”	0.546 (0.186-2.139)		
Item 13: “I have difficulty following my treatment when I am away from home or away from my routine (e.g., on weekends, traveling for work or on vacation)”	0.628 (0.380-4.533)		
Item 14: “I have support from family and/or friends who I can talk to at any time and who I can turn to for help”			0.511 (0.213-4.051)
Item 15: “I am afraid of the side effects of my medications”		0.500 (0.193-1.090)	
Item 16: “If I notice side effects related to my medications, I would talk to my doctor, pharmacist or nurse at the referral center about them as soon as possible”			0.519 (0.248-64.802)
Item 17: “If I notice side effects from my medications, I stop/would stop taking them or take/take less of the medications”	0.473 (0.164-2.050)		

95%CI: 95% confidence interval.

The latent G-H indices scores for subscales 1, 2, and 3 were 0.77, 0.81, and 0.66, respectively. For observed G-H, the scores were 0.59, 0.73, and 0.46 in the same sequence. The significant discrepancy between latent and observed G-H levels suggests instability in the model when applied to different population samples, limiting its generalizability.

The factor structure with the 17 items showed fit indices considered adequate (χ^2^ = 89.931, degrees of freedom (gl) = 88; p > 0.005; RMSEA = 0.010 (95%CI: 0.0000-0.0160); and RMSR = 0.07; CFI = 0.996; GFI = 0.940; AGFI = 0.907; NNFI and TLI = 0.995). The total questionnaire showed internal consistency, with Cronbach’s α and McDonald’s Ω registering 0.762 and 0.761 values, respectively. Table 4 shows the distribution of the variables according to their factor loadings in the three subscales.

## Discussion

This study describes the process of adapting and validating the ABQ-HIV to identify barriers to adherence to ART in PLHIV in Brazil. As a result, an adapted version comprising 17 items was obtained. The findings provided adequate evidence of content validity, the tool’s three subscales factorial structure, and its internal consistency, corroborating the questionnaire's reliability for the context in question.

This is the first study to report on the psychometric properties, reliability, and validity of the ABQ-HIV questionnaire in Brazil and internationally, expanding on the findings of the original study. A previous study only reported on the translation and adaptation process in Brazil ^35^. Initially, an attempt was made to analyze the factor structure of the questionnaire using an exploratory method. EFA was chosen to make up the set of preliminary analyses of the questionnaire, as it was also used in the original study ^18^.

The KMO index and Bartlett’s test of sphericity were adequate, indicating that the EFA was feasible. During the PA, it was decided to implement the EFA following the three subscales factorial structure already defined in the original version of the questionnaire, as established by Mueller et al. ^18^. However, it should be noted that several models were tested, and the results showed good psychometric quality, ensuring the generation of reliable psychometric indicators.

In the first PA (data not shown), the model revealed two subscales and recommended excluding two items: Item 2, on knowledge of medication, and Item 7, on adherence to medication at specific times or occasions. The possible association of these items with socially desirable responses may explain the result. However, the exclusion had no impact on the factor analysis indices or the quality of the measurement. Therefore, it was decided to keep all the items since the aim of the study was not to reduce the ABQ-HIV questionnaire.

In the three subscales model, an explained variance of 26.45%, 12.62%, and 8.69% was observed for each subscale extracted. As highlighted by Lorenzo-Seva & Ferrando ^31^, a higher explained variance indicates enhanced effectiveness of the subscales in elucidating the relationships among the original variables. Our findings align with the findings of Mueller et al. ^18^ in the development of the questionnaire, wherein the three subscales individually accounted for 22.1%, 8.9%, and 8.8% of the variance, respectively.

The fit of the model resulted in indicators aligned with the ideal values, meeting the criteria established in the specialized literature. The RMSEA was satisfactory, while the other indicators, CFI, GFI, and TLI, were above the desirable values (> 0.9) ^34^. The reliability of the questionnaire was estimated using the internal consistency method, using Cronbach’s α and McDonald’s Ω indices ^29^. Both coefficients showed acceptable results, higher than 0.70 and exceeding those found in the original study (α = 0.708) ^18^. It should be noted that these reliability coefficients would not increase if any items were eliminated.

The EFA then provided three subscales of the ABQ-HIV, which refer to unintentional adherence barriers, knowledge-related adherence barriers, and intentional adherence barriers. Factor loading was used to indicate how each item (variable) contributed to the formation of the factor. The results with three subscales provided satisfactory factor loadings ranging from 0.32 to 0.69, meeting recommended criteria ^36^. This was close to the findings of the original investigation, which ranged from 0.46 to 0.74 ^18^. However, the factor weights of the items varied between the two versions, which suggests cultural differences between the German and Brazilian contexts.

The “knowledge” subscale showed the best performance in terms of factor loadings, with adequate saturation levels. The factor loadings of items 2, 7, and 8, despite being the lowest in the investigation, remain within the adequate parameters established by Hair et al. ^37^. These authors propose values between 0.30 and 0.40 as adequate and above 0.50 as satisfactorily adequate. Therefore, the exclusion of these items from the model was not considered in this study.

Considering the three subscales proposed by the original questionnaire, the structure of the current construct showed a distribution among the items that differed from the original model ^18^. This result adds empirical data to the debate on the conceptual and functional differences of the subscales investigated. Other studies on the validation of questionnaires have also found that the subscales originally designed were not equivalent to the final subscales ^38^ ^,^ ^39^. In this sense, to verify the replicability of the final three subscales solution, it is recommended that future studies test this structure on new samples.

Given that the ABQ-HIV is currently undergoing its first phase of validation within the Brazilian context, the present study opted for EFA. This methodological choice is substantiated by its recommendation in circumstances where a robust empirical or conceptual foundation is lacking to inform the construction of the subscales model ^40^. The utilization of EFA aligns with best practices in psychometric questionnaire validation, particularly in scenarios characterized by a paucity of pre-established theoretical frameworks. This approach ensures a rigorous exploration of the underlying structure of the ABQ-HIV, laying the groundwork for its subsequent psychometric validation within the Brazilian population.

The absence of tailored questionnaires to assess adherence barriers among individuals with HIV/AIDS receiving ART in Brazil underscores a significant gap in understanding the complexities of adherence behaviours in this population. While existing validated instruments primarily focus on medication intake ^14^ ^,^ ^15^ ^,^ ^16^ ^,^ ^17^, they fail to delve into the nuanced barriers that may impede adherence. In this respect, the Brazilian version of the ABQ-HIV questionnaire is a potential tool for the early detection of patients at risk of non-adherence to ART, as it identifies potentially modifiable barriers, thereby facilitating physician-patient communication and identifying specific patient segments. The use of this tool can reduce readmissions, treatment failures, and costs for the healthcare system. Moreover, the results of the study could potentially contribute to more targeted and effective public health intervention policies and projects in the future. Although the socioeconomic and technical-scientific impacts of the study’s results are potential and are yet to be fully evaluated, they will potentially contribute to improving the quality of life of PLHIV and strengthening the health system’s response capacity. Essentially, the ABQ-HIV questionnaire can complement other available questionnaire options and be as effective as more expensive direct methods for measuring adherence.

This study has some limitations. The assessment of convergent and divergent validity for constructs associated with self-reported non-adherence was constrained by the unavailability of other questionnaires adapted to the Brazilian context. While acknowledging the absence of a universally recognized “gold standard” method for confirming medication non-adherence ^41^, future investigations could enhance the questionnaire’s validity by employing questionnaires already adapted for use in the Brazilian context ^15^ ^,^ ^16^. Additionally, exploring alternative measures, such as data derived from medication event monitoring systems, may present a more robust approach to validating the tool, albeit with associated resource demands ^18^. Furthermore, a substantial number of respondents comprised individuals with incomplete elementary school education, potentially influencing their comprehension of the questionnaire and its applicability due to their lower educational attainment. Notwithstanding these limitations, the outcomes of this investigation provide empirical support for the reliability and validity of the ABQ-HIV within the linguistic and cultural context of Portuguese as spoken in Brazil. Subsequent cross-cultural validations are needed to ascertain whether the questionnaire maintains its psychometric robustness when applied in varied national (including more cities and regions of Brazil) and/or international contexts.

In conclusion, the Brazilian adaptation of the ABQ-HIV has demonstrated its potential validity and reliability as a questionnaire for evaluating barriers to ART. The psychometric properties of the adapted version closely align with those of the original model, rendering it suitable for both clinical and research applications within the context of HIV infection in Brazil. The adaptation process was meticulous, incorporating qualitative and quantitative methods to ensure the objectivity, clarity, and relevance of the items while preserving their conceptual integrity. The resultant factor structure, comprising 17 items distributed across three subscales, exhibited adequacy by effectively explaining the observed variance and maintaining consistency throughout all validation phases, despite its configuration differing from the original model. This robust validation process underscores the utility of the Brazilian version of ABQ-HIV, providing a valuable tool for advancing our understanding and assessment of barriers to ART within the specific cultural and linguistic nuances of the Brazilian population.
