# Supplementary material for: Adaptation and validation of the Adherence Barriers Questionnaire for HIV Patients on Antiretroviral Therapy (ABQ-HIV) for the Brazilian context
Source: Cad Saude Publica. 2024 Sep 16;40(8):e00006324. doi: 10.1590/0102-311XEN006324 (PMC11405021; doi:10.1590/0102-311XEN006324)
Supplement: Supplementary file 1 [file 1678-4464-csp-40-08-EN006324-s.pdf]

Supplementary Material

Questionário de Barreiras de Adesão à Terapia Antirretroviral para Pessoas Vivendo com HIV (ABQ-HIV) versão brasileira (17 itens).

Por favor, marque o quanto as seguintes afirmações se aplicam a você.

		Aplica-se totalmente	Aplica-se em grande parte	Aplica-se pouco	Não se aplica
1	"Entendo bem o que meu médico(a), enfermeiro(a) ou farmacêutico(a) me explicou sobre o meu tratamento medicamentoso"	☐	☐	☐	☐
2	"Consigo citar os nomes dos meus medicamentos, para que servem e como devo ingerir"	☐	☐	☐	☐
3	"Confio no meu médico(a) e combino com ele(a) o meu plano de tratamento"	☐	☐	☐	☐
4	"Meus medicamentos só ajudam se eu os tomar de forma regular, como recomendado"	☐	☐	☐	☐
5	"Geralmente, qualquer medicamento é de alguma forma prejudicial e, portanto, você deve evitar tomar medicamentos sempre que possível"	☐	☐	☐	☐
6	"Em princípio, por não me sentir doente, às vezes não tenho certeza se deveria tomar meus medicamentos diariamente"	☐	☐	☐	☐
7	"Tomo meus medicamentos todos os dias automaticamente em um determinado horário ou em determinadas ocasiões (por ex., durante as refeições, na hora de dormir)"	☐	☐	☐	☐
8	"Gastos adicionais para ir receber meus medicamentos (por ex., transporte, alimentação fora de casa) representam uma verdadeira dificuldade para mim"	☐	☐	☐	☐
9	"Fico desconfortável quando outras pessoas percebem que estou tomando medicamentos"	☐	☐	☐	☐
10	"Frequentemente, esqueço coisas no meu dia a dia"	☐	☐	☐	☐
11	"Sinto-me abatido(a) com frequência; às vezes, também	☐	☐	☐	☐

	desanimado(a) e deprimido(a)”				
**12**	“Tenho dificuldades para tomar meus medicamentos (por ex., para engolir, dividir os comprimidos, abrir as embalagens) ou é difícil seguir as recomendações de uso dos medicamentos (por ex., em jejum, não ingerir determinados alimentos e/ou álcool)”	☐	☐	☐	☐
**13**	“Tenho dificuldade de seguir meu tratamento quando estou fora de casa ou da rotina (por ex., aos finais de semana, em viagens a trabalho ou nas férias)”	☐	☐	☐	☐
**14**	“Tenho apoio de familiares e/ou amigos com quem posso conversar a qualquer momento e a quem posso recorrer para pedir ajuda”	☐	☐	☐	☐
**15**	“Tenho medo dos efeitos colaterais dos meus medicamentos”	☐	☐	☐	☐
**16**	“Se noto/notasse efeitos colaterais relacionados aos meus medicamentos, converso/conversaria com meu médico(a), farmacêutico(a) ou enfermeiro(a) do centro de referência sobre eles o mais rápido possível”	☐	☐	☐	☐
**17**	“Se noto/notasse efeitos colaterais dos meus medicamentos, eu paro/pararia de tomá-los ou tomo/tomaria menos dos medicamentos”	☐	☐	☐	☐
